# Effects of Tongue Training Tasks on Intramuscular Activity Distribution: Evaluation Using Muscle Functional Magnetic Resonance Imaging

**DOI:** 10.1111/joor.70132

**Published:** 2025-12-08

**Authors:** Masahiro Sato, Satoshi Yamaguchi, Yoshinori Hattori

**Affiliations:** ^1^ Division of Aging and Geriatric Dentistry Tohoku University Graduate School of Dentistry Sendai Miyagi Japan

**Keywords:** deglutition disorders, humans, magnetic resonance imaging, rehabilitation, resistance training, tongue

## Abstract

**Background:**

From the perspective of site specificity, inducing muscle activity across a large area of the tongue through diversified tongue resistance training (TRT) may be an effective approach to improve tongue pressure (TP) and function. However, the distribution of muscle activity within the tongue during different TRT exercises remains unclear.

**Objectives:**

To examine the hypothesis that muscle activity distribution within the tongue varies with TRT direction using muscle functional magnetic resonance imaging (mfMRI), and to clarify the relationship between these distributions and functional outcomes.

**Methods:**

Twenty young volunteers were randomly assigned to either an elevation or a lateral 4 week TRT group. We performed mfMRI before (at rest) and immediately following each training task, before and after 4 weeks of TRT, and we measured maximal elevation, lateral TP, and oral diadochokinesis (ODK). A linear mixed‐effects model was used to analyse the functional outcomes.

**Results:**

After TRT, mfMRI revealed that elevation exercise activated the anterior tongue, including the origin of the genioglossus. Conversely, lateral exercise induced significant activity across a wider area extending to the tongue root. Post‐training maximal elevation TP and overall ODK values were significantly higher in the elevation training group. No significant differences between groups were observed in post‐training lateral TP.

**Conclusions:**

TRT direction changes the pattern of muscle activity in the tongue. This finding reveals qualitative aspects of TRT that cannot be evaluated by post‐training TP alone, offering a new perspective for designing more effective rehabilitation programs.

**Trial Registration:**

ClinicalTrials.gov identifier: UMIN000047658

## Graphical abstract

Patterns of muscle activity within the tongue vary by tongue resistance training direction. This finding will aid in designing more effective rehabilitation programs.

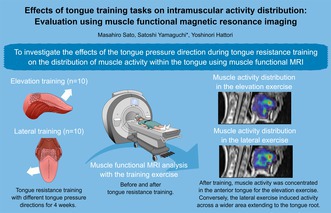

## 1 Introduction

The tongue is composed entirely of skeletal muscles and plays an important role in propelling food from the oral cavity to the pharynx during swallowing [1, 2]. However, aging decreases tongue muscle volume and strength, leading to a decline in swallowing function, which negatively affects the nutritional status of older adults [3]. Therefore, maintaining and improving tongue muscle strength may contribute to maintaining nutritional intake and extending healthy life expectancy in older populations [4, 5].

Tongue pressure (TP) serves as an indicator of tongue muscle strength, and tongue resistance training (TRT) effectively improves maximal TP (MTP) [6, 7, 8]. In physical therapy, TRT is categorised as muscle‐strengthening training (MST) [9]. Even when MST targets specific muscles, the areas of muscle hypertrophy differ according to the task or load [10]. Therefore, to determine effective training tasks for specific muscles, measuring coordinated muscle activity in addition to changes in muscle output is essential [11]. The tongue is a complex structure of multiple muscles [12]. Therefore, we hypothesise that varying the TRT task would alter the spatial distribution of intralingual muscle activity.

Previous research suggests that the site specificity of muscle activation during resistance training may cause uneven hypertrophy [13]. To achieve balanced hypertrophy throughout a muscle, eliciting activity across a wider area of the muscle is necessary. Furthermore, systematic variations in training tasks have been reported to be effective for improving muscle strength [14]. Accordingly, diversifying movement tasks to expand the areas of tongue muscle activity should be beneficial for TRT. However, in TRT research, insufficient information on muscle activity patterns and training effects of isometric exercises other than elevation tasks exists. Furthermore, although electromyography (EMG) analysis of the intrinsic tongue muscles has been previously reported [15, 16, 17], EMG cannot precisely identify the site of muscle activity derivation. Consequently, no reports that clearly show spatial differences in muscle activation between different tongue motor tasks are available.

Local activity in skeletal muscles can be measured using muscle functional magnetic resonance imaging (mfMRI) [18]. This technique is based on the principle that the transverse relaxation time (T2) within skeletal muscle is prolonged by exercise [19, 20], and the distribution of T2 changes reflects the distribution of glucose metabolism [21]. This allows for the quantitative evaluation of muscle activity in any region of the body [22].

Brain imaging methods enable the analysis of these data across participants. These included spatial normalisation, which involves aligning magnetic resonance (MR) images to a standard template [23], and statistical parametric mapping (SPM), which performs voxel‐wise statistical analysis [24]. By applying this method to mfMRI, we have previously confirmed that common activity distributions of the masticatory muscles can be statistically analysed [25]. This approach can be applied to the analysis of tongue muscle activity.

The purpose of this study was to clarify the differences in the spatial distribution of muscle activity during tongue elevation and lateral isometric movements using mfMRI. This represents the first step towards expanding the areas of tongue muscle activation through various training tasks to explore the possibility of establishing more effective TRT methods, and to correlate these spatial patterns with functional outcomes.

## 2 Materials and Methods

### 2.1 Research Ethics

The study's aims, methods, and safety were explained to all participants, and written informed consent was obtained. This study was approved by the Ethics Committee of Tohoku University Graduate School of Dentistry (approval no: 25396).

### 2.2 Participants

This study involved 20 healthy young volunteers without disorders of oral function (i.e., including tongue function and temporomandibular joint function), neuromuscular disorders, a history of orthodontic treatment, or contraindications to MRI. The participants were categorised into two groups of 10 volunteers. A one‐sided test was performed to detect the extension of T2 within the muscles associated with the specific exercise assigned to each group. In our previous study, the effect size (dz) of the T2 change in all masticatory muscles after clenching (unilateral clenching on the resin cap on the first molar, 40% of maximum bite force, for 1 min) was 0.86 [26, 27]. On the basis of an effect size of dz. = 0.86 and an error probability of α = 0.05, the sample size required to achieve a statistical power of 1‐*β* = 0.8 for a one‐sided test was 10 (G*Power3; Heinrich Heine University, Düsseldorf, Germany) [28].

### 2.3 TP Measurement Devices

TRT was performed for all participants using a TP measurement device (TP‐02; JMS Corporation, Tokyo, Japan) [29].

**FIGURE 1:**
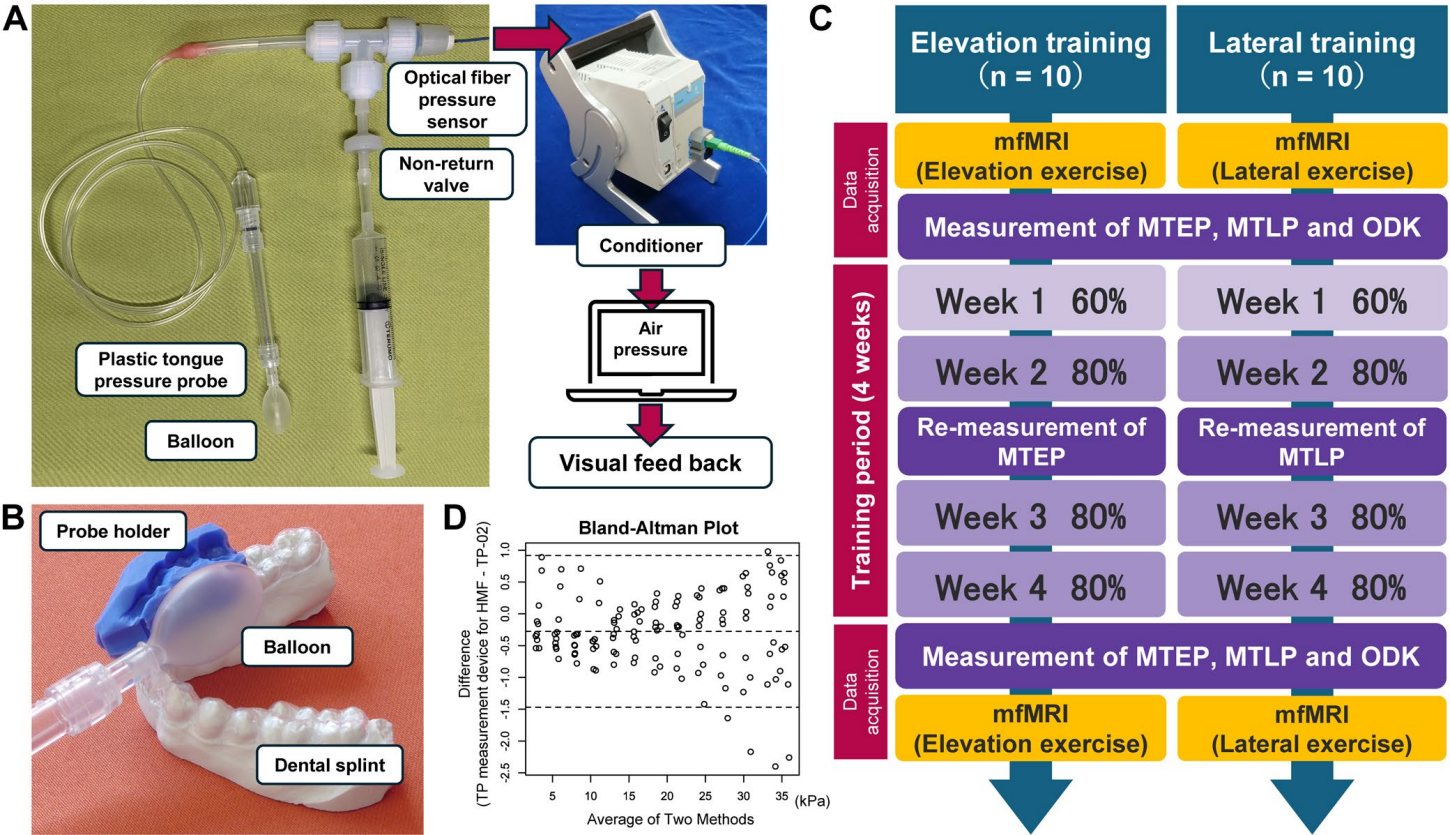
Overview of the equipment and materials used in this study and the research workflow. (A) Overview of the tongue pressure measurement device for high magnetic fields and the experimental setup for visual feedback. (B) Overview of the probe holder and dental splint for lateral training. (C) The flow diagram of the study. (D) Bland–Altman plot of the TP measurement device for HMF and the TP‐02 measurement values. TP: Tongue pressure; HMF: High magnetic field.

Accurate T2 measurements require an MRI scan performed immediately after task completion because exercise‐induced T2 changes resolve rapidly. Precision equipment containing metal parts cannot be used in the scanner room because an MR scanner generates a high magnetic field (HMF). Therefore, we constructed an original TP measurement device for the HMF by applying an optical fibre pressure sensor (FOP‐M‐BA; FISO Technologies Inc., Québec City, QC, Canada) connected to a plastic TP probe (Figure 1A). Using a fibre‐optic cable, the pressure sensor was connected to a conditioner (EVO‐SD‐2; Fiso Technologies Inc.) in the operations room outside the scanner room. We observed the change in air pressure caused by pressing the balloon of the TP probe in real time using software on a personal computer connected to the conditioner.

To assess the consistency between the air pressure measured by the TP measurement device for HMF and the TP measured by the TP‐02, a force gauge (ZP‐1000 N; Imada Corporation, Aichi, Japan) was used to apply the same external force to both devices, 10 times, each at 13 different force levels. Agreement between the TP measurement device for HMF and the TP‐02 was assessed using a Bland–Altman plot, and the intraclass correlation coefficient (ICC) was calculated.

### 2.4 TRT

We randomly assigned each participant to one of two isometric training conditions: “elevation training” and “lateral training”. Elevation training was performed by applying isometric pressure to the balloon probe of the TP‐02 positioned at a distance of 10 mm behind the tongue apex against the hard palate. Lateral training was performed by isometric pressing the balloon probe positioned on the right premolar against a dental splint and probe holder (described below). To examine the differences between the ipsilateral and contralateral sides in the distribution of tongue muscle activity during lateral movement, only isometric movement to the right side was used as the task.

In both training groups, participants pressed their tongue against the probe for 3 s at 60%–80% of the MTP. This was performed 10 times in each set, with 3 sets performed daily. A 1 min rest was applied between sets. The training was conducted on three non‐consecutive days per week over a period of 4 weeks [8]. The training period in the cited study was 8 weeks; however, our preliminary experiment demonstrated that a period of 4 weeks was sufficient to achieve a significant increase in MTP. Consequently, in consideration of the burden on participants, the training period was modified to 4 weeks. The MTP of each participant was measured using the TP‐02 before starting each training program. Participants were instructed to hold the TP‐02 balloon probe in the specified position in their mouth, as described above, and press it with their tongue at maximum force for 3 s. This measurement was recorded three times, and the average value was used as the MTP. During TRT, the TP was set at 60% of the MTP for the first week and 80% from the second week onward to ensure participant safety. At the end of the second week, the MTP was measured again, and the load was reset. TP was maintained at 80% of the reset MTP during the third and fourth weeks [30]. A TP‐02 was provided to each participant and all training was conducted at the participants' homes. The participants monitored their tongue pressure displayed in real time on the TP‐02 and performed training at the specified tongue pressure based on visual feedback. Adherence to the home‐based training program was monitored using a communication system established through the LINE messaging application (LY Corporation, Tokyo, Japan). Participants reported the completion of 1 day of training via text messages, which were regularly monitored and the information was recorded by the research team. The adherence rate was calculated by dividing the number of training days by 12 (total number of training days) and multiplying the result by 100.

### 2.5 Probe Holder for Lateral Training

(Figure 1B) shows the probe holder and dental splint. The holder was used for lateral TP measurements and lateral training. A bite block made of silicone putty (Imprinsis Putty; Tokuyama Dental Corporation, Tokyo, Japan) was fixed to the mandibular dental splint made of thermoplastic resin. The bite block was given a concave shape in the area corresponding to the right premolar to standardise the positioning of the probe during training and measurement.

### 2.6 Measurement Items

For each participant, maximal tongue elevation pressure (MTEP), maximal tongue lateral pressure (MTLP), oral diadochokinesis (ODK) were measured at the time described in the “Research flow” section below. Furthermore, mfMRI was conducted before and after the participants performed the same task exercise as the training task.

MTP of the elevation or lateral training tasks was measured three times, and the average was used as the MTEP or MTLP. In the early stages of MST, strength gains are thought to be largely the result of nervous system adaptations and improved coordination between muscles, rather than muscle hypertrophy, and this may lead to improved motor skills [31]. In this study, ODK was measured as an indicator to observe changes in tongue motor function resulting from TRT. The participants were asked to repeat the <pa>, <ta>, and <ka> sounds as quickly as possible for 5 s. The total number of repetitions was counted using the Kenkoukun Handy (TKK‐3351; Takei Kiki Kogyo Co. Ltd., Niigata, Japan), and the number of repetitions per second was recorded.

We performed all mfMRI scans using a G‐scan brio (0.25 T) (Esaote, Genoa, Italy) with a neck coil. The participants were placed in the dorsal position and earplugs were worn to reduce noise during imaging. The spin echo sequence parameters were as follows: repetition time (TR) = 2800 ms; echo time (TE) = 28/90 ms; flip angle = 90°; 17 slices, slice thickness = 5 mm; slice gap = 0 mm; matrix = 256 × 256; field of view = 300 nm; pixel size = 1.17 × 1.17 mm; and scan time = 6 min 7 s. After MRI at rest, the participants performed the same exercise task as the training task in the MR gantry using a TP measurement device for HMF. The measured TP value was displayed on a screen in the scanner room using a projector and fed back through a mirror attached to a neck coil to maintain the exercise intensity. MR images were captured immediately after the exercise task.

### 2.7 Research Flow

(Figure 1C) shows a flow diagram of the study. Initially, the MTEP, MTLP, and ODK for all participants were measured, and mfMRI was conducted before and after the participants performed the same task exercise as the training task. After 4 weeks of training, mfMRI was repeated, and MTEP, MTLP, and ODK were measured.

### 2.8 Image Analysis

**FIGURE 2:**
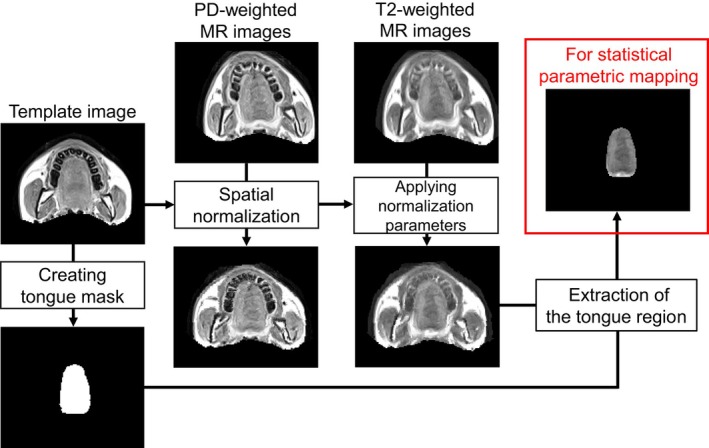
Pipeline of the spatial normalisation of the T2‐weighted MR image to the template image and extraction of the tongue regions with a mask image. The transformation parameter for performing nonlinear registration of the proton density‐weighted MR image to the template image is applied to the corresponding T2‐weighted MR images. MR: Magnetic resonance.

Functional brain imaging analysis involves the integration and averaging of brain activity distribution within each participant group. Two techniques are utilised to achieve this and to enable comparisons between the groups. First, spatial normalisation is used to align the brain morphology in magnetic resonance (MR) images obtained from multiple participants to a standard template, thereby standardising the spatial distribution for group‐level analysis [23]. Subsequently, statistical parametric mapping (SPM) performs voxel‐wise statistical analysis of these normalised MR images to compare activities between groups [24]. We applied this method to the mfMRI analysis.

(Figure 2) shows an overview of image analysis. In mfMRI analysis, the T2 values for all voxels are usually calculated from the MR images and are used as an index of muscle activity. However, analysis using the signal intensity of T2‐weighted MR images has also been reported [19, 32, 33]. The signal intensity of the T2‐weighted MR images was directly used for statistical mapping analysis because the calculated T2 values often deviated from the established range on the 0.25‐T MR system used in this study.

From the proton density (PD)‐weighted MR images (TE: 28 ms; TR: 2800 ms) of all participants, the image exhibiting the most typical head and neck morphology with the least degree of tilt was selected. Only the region of the lower face was extracted from this image and used as a template. Mask images were created for all PD‐weighted MR images of all participants to extract only the region of the lower face. Each mask image was applied to the PD‐ and T2‐weighted MR images (TE: 90 ms; TR: 2800 ms). The spatial normalisation of the PD‐weighted MR images to the template image was performed using Advanced Normalisation Tools [34]. After confirming the spatial normalisation accuracy, the same transformations were applied to the T2‐weighted MR images. A mask image was created to extract only the tongue region from the template. The same mask image was applied to all normalised T2‐weighted MR images to create spatially normalised T2‐weighted MR images of the tongue. After excluding voxels with signal intensities below “0”, spatial smoothing was performed using a 6‐mm full‐width at half‐maximum Gaussian filter [25].

Using SPM 12 software (Wellcome Institute, London, UK) [24], a voxel‐wise comparison of the signal intensity at rest and immediately after elevation or lateral exercises was performed using a paired *t*‐test. The tongue in the MR images used in this study contained more than 9000 voxels. To address the issue of multiple comparisons across thousands of voxels, we applied a false discovery rate (FDR) correction with a threshold of *p* < 0.05 [35]. This procedure controls for the expected proportion of false positive results among the clusters of voxels identified as significant, thereby ensuring that the reported findings are robust and not merely due to chance. The resulting statistical maps were then thresholded, and clusters were considered significant if they survived this correction. The voxel‐wise maps of the effect size *d* were created from the T‐value image generated by SPM analysis. When calculating *d*, the following formula—an approximate equation that can be used for comparisons between two independent groups of the same sample size—was applied in relation to each voxel: Cohen's *d* = t/√n [36, 37], where t represents the voxel values of the T‐value images, and n represents the number of participants. Finally, a voxel‐wise analysis of variance (ANOVA) with FDR correction was performed to compare the changes in signal intensity between the elevation and lateral exercises.

### 2.9 Statistical Analysis

Linear mixed‐effects models (LMMs) were used to analyse MTP and ODK, which are continuous dependent variables of the primary outcome measure. The models were used to examine the effects of the between‐subjects factor (TRT type) and the within‐subjects factors (Time point and MTP type, or Time point and ODK type). The LMM model on MTP included TRT type (elevation training vs. lateral training), Time point (Pre‐ vs. Post‐training), MTP type (MTEP vs. MTLP), and their interactions as fixed effects (MTP model). The LMM model on ODK included TRT type, Time point, ODK type (<pa> vs <ta> vs <ka>), and their interactions as fixed effects (ODK model). To control for Pre‐training differences between the participants, the baseline MTP (Pre‐training MTP) or baseline ODK (Pre‐training ODK) was included as a covariate in the MTP and ODK models, respectively. To account for the non‐independence of repeated measures from the same participants, participant identity (ID) was included as a random intercept. When a significant three‐way interaction was detected, simple effects analysis was performed to further investigate the differences between the two groups. When only the two‐way interaction was significant, post hoc analyses were performed on the estimated marginal means, pooling the levels of factors not included in that interaction. Tukey's multiple comparison correction was applied to the pairwise comparisons. For the ODK model, when the two‐way interaction between TRT type and Time Point was found to be significant, whereas the three‐way interaction remained non‐significant, an additional exploratory analysis was performed. This involved examining the pairwise comparisons of the TRT type × Time Point interaction within each ODK type (<pa>, <ta>, <ka>) to explore potential differences in training effects among the ODK types. The results from this exploratory analysis were interpreted with caution owing to the non‐significant three‐way interaction. All statistical analyses were performed using R statistical software (version 4.5.1; R Foundation for Statistical Computing, Vienna, Austria). The LMM was implemented using the “lme4” and “lmerTest” packages, and post hoc analyses were conducted using the “emmeans” package. A significance level of *p* < 0.05 was applied to all statistical tests.

## 3 Results

### 3.1 Demographic Characteristics

The mean age of study participants was 27.8 ± 2.4 years, and 35.0% were female. All participants were Japanese, except for one female participant of Middle Eastern descent.

### 3.2 Adherence of the Home‐Based Training Program

The adherence rate for the elevation training group and the lateral training group was 68.3% ± 14.1% and 74.2% ± 12.7%, respectively.

### 3.3 Consistency Between the TP Measurement Device for HMF and TP‐02 Measurements

(Figure 1D) shows the Bland–Altman plot of the TP measurement device for HMF and the TP‐02 measurement values. The ICC for single measurements was 1.00 (95% CI: 1.00–1.00, *p* < 0.001), indicating excellent agreement between the two methods.

### 3.4 LMM on MTP and ODK

(Table 1) shows the MTEP, MTLP, and ODK values of all the participants before and after training. The MTP model revealed a significant three‐way interaction between TRT type, Time point, and MTP type, *F*(1, 52.53) = 11.01, *p* = 0.002 (Table 2). This suggests that the effect of the TRT type on MTP varies according to both the Time point and the specific type of MTP. The model also indicated a significant two‐way interaction between TRT type and MTP type, *F*(1, 52.77) = 11.58, *p* = 0.001. A significant main effect of Time point was observed, *F*(1, 52.53) = 110.23, *p* < 0.001, indicating that MTP significantly increased from Pre‐ to Post‐training across all groups. Baseline MTP, included as a covariate, was a significant predictor of MTP values across all Time points, *F*(1, 31.55) = 178.66, *p* < 0.001.

**TABLE 1:** Pre‐ and Post‐training maximal tongue pressure and oral diadochokinesis.

Training	Measurement item		Pre‐training	Post‐training
Elevation training	MTP (kPa)	MTEP	36.79 (7.08)	50.13 (10.16)
		MTLP	22.41 (5.54)	28.60 (6.57)
	ODK (times/s)	<pa>	6.66 (1.41)	6.68 (1.39)
		<ta>	6.98 (1.06)	7.56 (0.67)
		<ka>	6.48 (1.56)	7.04 (0.44)
Lateral training	MTP (kPa)	MTEP	41.24 (9.42)	47.20 (8.85)
		MTLP	28.26 (7.74)	38.30 (6.97)
	ODK (times/s)	<pa>	6.74 (0.58)	6.62 (0.53)
		<ta>	6.96 (0.64)	6.82 (0.67)
		<ka>	6.40 (0.81)	6.56 (0.62)

*Note:* Mean (standard deviation).

Abbreviations: MLTP, maximal tongue lateral pressure; MTEP, maximal tongue elevation pressure; MTP, maximal tongue pressure; ODK, Oral diadochokinesis.

**TABLE 2:** Summary of fixed effects from the linear mixed model analysis on maximal tongue pressure.

Model	Effect	df1	df2	F	*p*
MTP model	Baseline MTP	1	31.55	178.66	< 0.001
	TRT type	1	17.26	0.093	0.764
	Time point	1	52.53	110.23	< 0.001
	MTP type	1	64.14	3.28	0.075
	TRT type × Time point	1	52.53	1.09	0.302
	TRT type × MTP type	1	52.77	11.58	0.001
	Time point × MTP type	1	52.53	0.82	0.368
	TRT type × Time point × MTP type	1	52.53	11.01	0.002
ODK model	Baseline ODK	1	24.85	183.45	< 0.001
	TRT type	1	17.31	3.92	0.064
	Time point	1	89.97	3.01	0.086
	ODK type	1	93.26	1.50	0.229
	TRT type × Time point	1	89.97	4.26	0.042
	TRT type × ODK type	1	90.05	0.83	0.441
	Time point × ODK type	1	89.97	1.40	0.253
	TRT type × Time point × ODK type	1	89.97	0.68	0.510

Abbreviations: Df, degree of freedom; MTP, maximal tongue pressure; ODK, Oral diadochokinesis; TRT, tongue resistance training.

**FIGURE 3:**
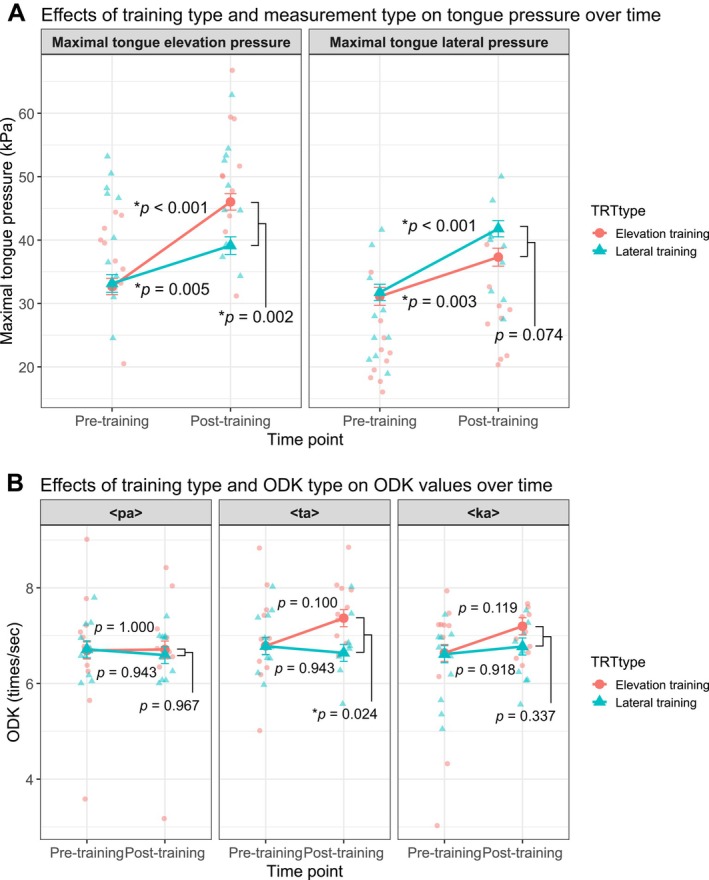
(A) Results of a simple effects analysis of training type and measurement type on tongue pressure over time. (B) Results of a simple effects analysis of training type and ODK type on ODK values over time. *P*‐values indicate the results of pairwise comparisons from the simple effects analysis based on a linear mixed‐effects model. ODK: Oral diadochokinesis. *p < 0.05.

Simple effects analysis revealed a significant effect of TRT type on MTEP after training (Figure 3A). Elevation training resulted in significantly higher MTEP than Lateral training at the Post‐training Time point (*t* = 3.83, *p* = 0.002). Furthermore, both Elevation training (*t* = 7.88, *p* < 0.001) and Lateral training (*t* = 3.52, *p* = 0.005) led to a significant increase in MTEP from Pre‐ to Post‐training. No significant difference in MTEP was found between the two training groups at baseline (*t* = −0.27, *p* = 0.993). Conversely, the post‐training MTLP was not significantly different between the Elevation training and Lateral training groups (*t* = −2.47, *p* = 0.074). However, both Elevation training (*t* = 3.66, *p* = 0.003) and Lateral training (*t* = 5.93, *p* < 0.001) were found to be effective in significantly increasing MTLP from Pre‐ to Post‐training, and the mean change in the Lateral training group (estimate = 10.04) was numerically larger than that in the Elevation training group (estimate = 6.19).

The ODK model revealed a significant two‐way interaction between TRT type and Time point, *F*(1, 89.97) = 4.26, *p* = 0.042, whereas the three‐way interaction involving ODK types was not significant, *p* = 0.510 (Table 2). Baseline ODK, included as a covariate, was a significant predictor of ODK values across all Time points, *F*(1, 24.85) = 183.45, *p* < 0.001.

Post hoc pairwise comparisons were conducted on the estimated marginal means averaged over the ODK type factor. The results indicated a specific training effect for the Elevation training group. The Elevation training group demonstrated a significant increase in the overall ODK value from Pre‐ to Post‐training (Estimated Difference = 0.39 times/s, *p* = 0.042). The mean ODK value for the Elevation group increased from 6.70 times/s (Pre‐training) to 7.09 times/s (Post‐training). Furthermore, the overall ODK value for the Elevation training Post‐training group was significantly higher than that of the Lateral training Post‐training group (Estimated Difference = 0.42 times/s, *p* = 0.030). In contrast, the Lateral training group showed no significant change from Pre‐ to Post‐training (*p* = 0.996; mean change: −0.03 times/s), and baseline ODK values were confirmed to be identical between the two groups (*p* = 1.000). Exploratory analysis of the ODK subtypes revealed a statistically significant difference only for the <ta> sound (Figure 3B). Particularly, the <ta> ODK value for the Elevation training Post‐training group was significantly higher than that of the Lateral training Post‐training group (estimated difference = 0.726 times/s, *p* = 0.024), suggesting a specific training benefit of elevation exercise for the <ta> motor task.

### 3.5 Changes in Signal Intensity of T2‐Weighted MR Image Before and After Tongue Exercise

**FIGURE 4:**
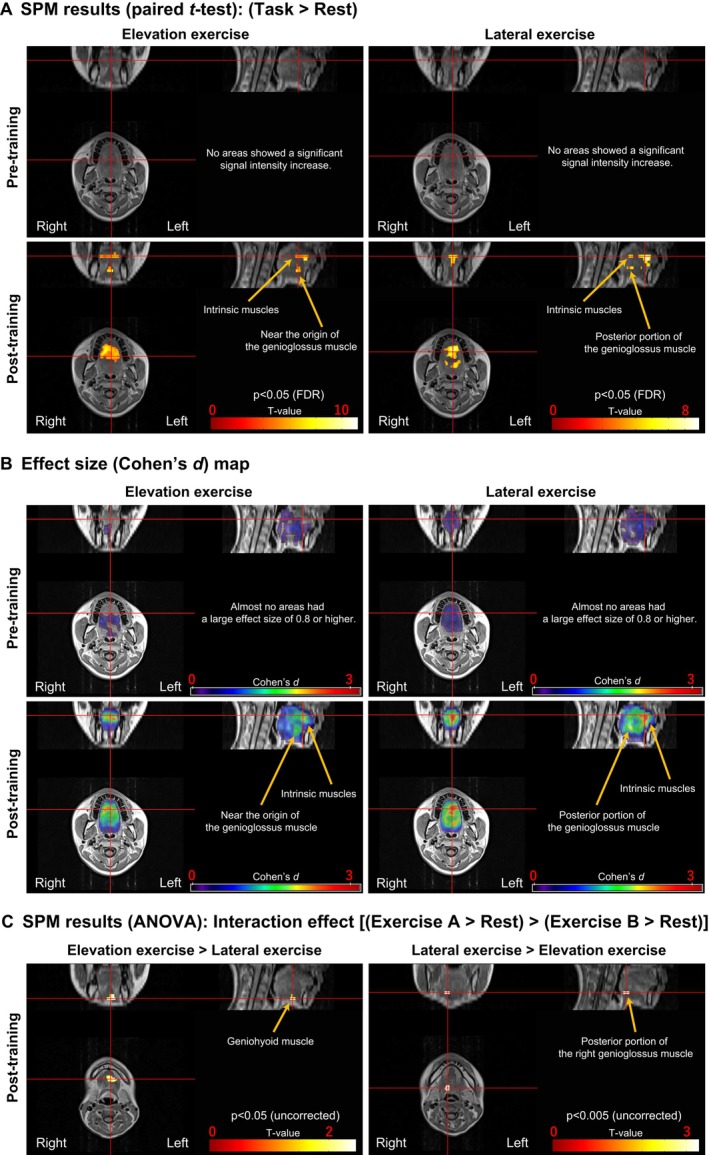
Results of MR image analysis. (A) Statistical parametric maps (cluster‐corrected p [FDR] < 0.05) of the voxels show significantly increased signal intensity in T2‐weighted MR images after exercises. The colour bar represents the *t*‐value. At Pre‐training, no regions of significantly increased signal intensity were detected. At Post‐training, intrinsic muscles and the area near the origin of the genioglossus muscle exhibited significantly increased signal intensity after the elevation tongue exercise. Conversely, the intrinsic muscles and the posterior portion of the genioglossus muscle exhibited significantly increased signal intensity after the lateral tongue exercise. (B) Effect size (Cohen's d) maps of signal intensity increase in T2‐weighted MR images. The colour bar represents the effect size (Cohen's d). Pre‐training, almost no areas with an effect size of ≥ 0.8 (a large effect size) were observed. Post‐training, for the elevation exercise, a large effect size was concentrated in the anterior region of the tongue. For the lateral exercise, a large effect size was observed over a wider area extending from the anterior region to the tongue root. (C) Statistical parametric maps of the voxels show increased signal intensity in T2‐weighted MR images for each exercise compared with the others (*p* < 0.05, uncorrected for multiple comparisons). The colour bar represents the *t‐*value. Greater increases in signal intensity were observed near the geniohyoid muscle after the elevated exercise (*p* = 0.01) and in the right tongue root (posterior portion of the right genioglossus muscle) after the lateral exercise (*p* = 0.001). MR: Magnetic resonance.

Figure 4A shows the results of SMP analysis comparing signal intensity after each motor task to resting‐state signal intensity (FDR cluster‐corrected, *p* < 0.05). Before TRT, no regions of significantly increased signal intensity were detected after the elevation and lateral tongue exercises, compared with the resting state. After TRT, the dorsum of the tongue (intrinsic muscles) and the area near the origin of the genioglossus muscle exhibited significantly increased signal intensity after the elevation tongue exercise. Conversely, the dorsum (intrinsic muscles) and root of the tongue (posterior portion of the genioglossus muscle) exhibited significantly increased signal intensity after the lateral tongue exercise, compared with the resting state.

### 3.6 Effect Size Map

(Figure 4B) shows the effect size (Cohen's *d*) maps for the elevation and lateral tongue exercises Pre‐ and Post‐training. Cohen's *d* values of ≥ 0.8 are generally considered a large effect size [37]. Pre‐training, almost no areas with an effect size of ≥ 0.8 were observed. Post‐training, for the elevation exercise, a large effect size was concentrated in the anterior region of the tongue. For the lateral exercise, a large effect size was observed over a wider area extending from the anterior region to the tongue root.

### 3.7 Differences in the Region of Increased Signal Intensity Between the Elevation and Lateral Tongue Exercises

(Figure 4C) shows regions with a differential Post‐training signal increase between the two exercise types. Specifically, it identifies areas where the increase from the elevation exercise was greater than that from the lateral exercise (Elevation > Lateral) and areas where the opposite occurred (Lateral > Elevation) (*p* < 0.05, uncorrected for multiple comparisons). Greater increases in signal intensity were observed near the geniohyoid muscle after the elevated exercise (*p* = 0.01) and in the right tongue root, specifically, the posterior portion of the right genioglossus muscle, after the lateral exercise (*p* = 0.001). However, these increases were not significant after correction for multiple comparisons (FDR cluster‐corrected, *p* < 0.05).

## 4 Discussion

To our knowledge, this is the first report of tongue muscle activity distribution using spatial normalisation and SPM. EMG is the gold standard for measuring muscle activity. EMG analyses for intrinsic tongue muscle activity using inserted electrodes as well as muscle activity analyses in the posterior part of the tongue using surface EMG from the neck have been reported [38]. However, capturing the three‐dimensional distribution of muscle activity throughout the tongue using EMG is challenging. The method used in this study allowed us to map the three‐dimensional distribution of muscle activity within the tongue.

The low adherence rate for the training protocol in this study (68.3% ± 14.1% for elevation training and 74.2% ± 12.7% for lateral training) suggests that the analysis results are unlikely to reflect the maximum training effect. However, analysis using LMM showed that the fixed effect of Time point was significant. Both the MTEP and MTLP increased significantly in the elevation and lateral training groups (Table 2, Figure 3A). This result, consistent with previous studies demonstrating the effectiveness of tongue training [39], suggests that even with a lower‐than‐planned adherence rate, the intervention was robust enough to induce a measurable training effect. Therefore, we believe that sufficient changes were induced to allow for a meaningful analysis of the differences in muscle activity distribution within the tongue.

The high ICC for single measurements between the two devices indicates that the TP measurement device for use in HMFs shows a high level of agreement with the TP‐02, the current gold standard in Japan. Although a tendency for measurements to be slightly lower than the TP‐02 at higher pressures in the Bland–Altman plot exists (Figure 1D), the two devices are compatible. Therefore, using the TP measurement device for HMF is considered appropriate.

The three‐way interaction revealed by the MTP model was significant. Simple effect analysis revealed that elevation training had a significantly greater effect on MTEP than lateral training. These results support previous findings that elevation training effectively increases tongue elevation pressure [6]. Regarding MTLP, a trend suggesting greater effectiveness of lateral training compared to elevation training was observed, although the difference was not significant (*p* = 0.074). In future studies, a larger sample size may be required to detect significant effects; however, the results of the present study did not show any specific effects of lateral training on MTLP (Figure 3A).

The ODK model showed a significant TRT type × Time Point interaction. The Elevation training group demonstrated a significant increase in overall ODK value and a significantly higher Post‐training value compared to the Lateral training group. This suggests that the adaptation pattern induced by the elevation task effectively enhanced the neural efficiency and coordination required for general ODK performance.

LMM revealed that baseline MTP and ODK were significant covariates, confirming that Pre‐training values strongly influenced outcomes. Incorporating these covariates ensured that training effects were appropriately adjusted for Pre‐existing individual differences.

In the mfMRI before TRT, no regions of significantly increased signal intensity were detected after elevation and lateral tongue exercises, compared with the resting state (Figure 4A). However, regions with significantly increased signal intensity were detected after TRT. The SPM of signal intensity represents the region of significantly high activity within the tongue muscle, which was common to all participants. Before TRT, the areas of muscle activity during tongue movement likely varied among participants. In the initial stages of resistance training, muscle strength increases owing to neural adaptation and improved muscle coordination rather than muscle hypertrophy [31]. The participants may have learned more efficient muscle activities for performing their exercise tasks during the 4 weeks of TRT. This may have resulted in the convergence of muscle activity sites to areas common to all participants. Post‐TRT mfMRI detected significant muscle activity in the dorsum of the tongue (intrinsic muscles) and in the area near the origin of the genioglossus muscle during the elevation exercise, and in the dorsum (intrinsic muscles) and root of the tongue (posterior portion of the genioglossus muscle) during the lateral exercise (Figure 4A).

It has been suggested that the genioglossus muscle is functionally divided into five internal segments for precise partial control [40]. The results of this study suggest that the active site of the genioglossus muscle varies depending on the direction of TRT.

The effect‐size map obtained after training showed strong activity near the origin of the genioglossus muscle and in the intrinsic muscles of the tongue during elevation exercise, consistent with the SPM results (Figure 4B). Previous research has also suggested that tongue protrusion with resistance requires activation of the genioglossus muscle and the intrinsic tongue muscles [15]. This likely reflects a functional division, whereby the genioglossus muscle acts as the main force pulling the tongue forwards and upwards, while the intrinsic tongue muscles shape the tip of the tongue precisely and regulate its rigidity. In contrast, the area of muscle activity was concentrated in the anterior part of the tongue during the elevation exercise, whereas a wider range of muscles was active during the lateral exercise, with minimal difference between the left and right sides despite the movement being performed on the right side only (Figure 4B). A finite element model suggests that lateral flexion of the tongue causes contraction of the muscles on the ipsilateral side, as well as contraction of the interior contralateral muscles of the tongue [41]. Moreover, the tongue, a muscular hydrostat, stiffens throughout its entire structure through extensive muscle contraction to maintain structural stability against external forces [42, 43], when the probe is pressed against the lateral border of the tongue. Our findings may reflect these muscle activities.

(Figure 4C) suggests a trend whereby the tongue root is more engaged during lateral exercises compared with elevation exercises, although this becomes non‐significant after multiple comparisons correction. Muscle activity increases in the posterior tongue during the mastication of solid foods, highlighting the importance of this activity for bolus formation and movement [44]. Furthermore, our results are consistent with those of previous studies that lateral movement of the tongue contributes to bolus formation [45]. In contrast, the activity of the geniohyoid muscle may be stronger during elevation exercises than during lateral exercises (though it becomes non‐significant after multiple comparison corrections). Reportedly, tongue elevation training increases muscle activity and thickness of the suprahyoid muscle group [46]. In this study, the ODK values improvement in the elevation training group was primarily driven by <ta> (Figure 3B). This aligns with previous studies reporting ODK improvements [47]. The geniohyoid muscle may be strongly involved in <ta> pronunciation. This phenomenon might be the result of neural adaptation and enhanced muscle coordination during the early stages of resistance training [31]. Previous studies have reported that TRT also has motor learning effects [48], and induces neuroplasticity in corticomotor control of the tongue [49, 50].

As mentioned previously, a comparison of the Post‐training MTP and ODK using LMM did not confirm a task‐specific functional superiority for lateral TRT, showing no effect on overall ODK. However, the mfMRI results may suggest that, compared with elevation exercise, lateral tongue exercise induces a wider range of muscle activity and is particularly effective for training the root of the tongue. The results of this study demonstrated that TRT direction changes the pattern of muscle activity in the tongue. This finding reveals qualitative aspects of TRT that cannot be evaluated by Post‐training TP alone, offering a new perspective for designing more effective rehabilitation programs. By analysing muscle activity distribution during actual functions, such as swallowing, using mfMRI, it may be possible to develop TRT using exercise tasks that are optimised for the recovery of specific functions.

This study has some limitations. First, the short duration of and low adherence rate to the training protocol may have limited the ability of this study to accurately assess its true effects. Increasing the load of the training protocol and improving adherence may reveal a significant trend towards higher MTLP after lateral training than after lifting training. The reliability of the conclusions drawn from these data is limited. Second, we used a 0.25‐T MRI scanner. Thus, the low signal‐to‐noise ratio and resolution of the 0.25‐T MRI scanner, compared to ≥ 1.5 T, may have limited the accuracy of our analysis. If high‐resolution images are acquired using an MRI scanner of ≥ 1.5 T, it may be possible to analyse more detailed areas of muscle activity and localised muscle volume changes in the tongue due to training.

## 5 Conclusion

The distribution of muscle activity within the tongue can be evaluated by applying spatial normalisation and SPM to the mfMRI analysis, which was not feasible using previous methods. The analysis results indicate that the muscles affected by lateral TRT may differ from those affected by elevation TRT. The main finding of this study is the visualisation of differences in muscle activity patterns following different training tasks. We did not conclude that one training method is functionally superior to the other. Future research should be focused on the distribution of muscle activity within the tongue during various oral functions to further elucidate the clinical significance of the present findings.

## Author Contributions

Masahiro Sato: contributed to design, data acquisition, interpretation, and statistical analyses, and drafted and critically revised the manuscript. Satoshi Yamaguchi: contributed to the conception, design, data acquisition, and interpretation; performed all image analyses; drafted and critically revised the manuscript. Yoshinori Hattori: contributed to the conception and design of the study and critically revised the manuscript. All authors gave final approval and agreed to be accountable for all aspects of this work.

## Funding

This work was supported by Japan Society for the Promotion of Science, 18K09675, 21K09970.

## Conflicts of Interest

The authors declare no conflicts of interest.

## Data Availability

The data that support the findings of this study are available from the corresponding author upon reasonable request.
